# Ankle dorsiflexion range of motion and landing postures during a soccer-specific task

**DOI:** 10.1371/journal.pone.0283150

**Published:** 2023-03-16

**Authors:** Hadi Akbari, Yohei Shimokochi, Bahram Sheikhi

**Affiliations:** 1 Department of Sport Sciences, University of Zabol, Zabol, Iran; 2 Department of Health and Sport Management, School of Health and Sport Sciences, Osaka University of Health and Sport Sciences, Osaka, Japan; 3 Department of Biomechanics and Sport Injuries, Kharazmi University, Tehran, Iran; Universita degli Studi di Milano, ITALY

## Abstract

**Introduction:**

Ankle dorsiflexion range of motion (DF-ROM) has been shown to be associated with poor landing posture. However, previously used tasks have been controlled, and it is unclear whether clinical measurements of the ankle DF-ROM, are associated with landing positions during sport-specific task. This study sought to determine the relationship between ankle DF-ROM and landing positions.

**Methods:**

Thirty male soccer players participated in this study. The ankle DF-ROM was measured by the weight bearing lunge test in degrees using a cell phone app (TiltMeter). Landing patterns were assessed during a soccer-specific task using landing error scoring system items using Kinovea software. Simple correlations were used to evaluate the relationships between ankle DF-ROM and landing error scores.

**Results:**

Significant correlations were found between ankle DF-ROM and landing errors (*r* = -0.450, P = 0.006). A decreased ankle DF-ROM was associated with greater landing errors in a soccer specific situation.

**Conclusion:**

These results suggest that ankle DF-ROM may serve a useful clinical measure for identifying poor landing posture in the real-world environment. Therefore, assessment of ankle DF-ROM could be included in the screening process, which could help identify the cause of the faulty motion.

## Introduction

Lower limb injury prevention appears to be critical for soccer players because greater number of these injuries were reported in soccer than in field hockey, volleyball, handball, rugby, basketball, cricket, and badminton [1]. Overall, 50–80% of lower limb injuries in soccer are ankle sprains and knee ligament tears, with anterior cruciate ligament (ACL) injury being a serious injury with a high occurrence rate [1, 2]. While majority of ACL injuries in soccer are non-contact, such as during landing from jump [3, 4], implementing ACL injury prevention training to modify lower limb joints neuromusclar functions has shown to be effective to reduce noncontact ACL injury occurrence rate in soccer [5]. Yet, it is crucial to identify modifiable ACL injury risk factors that may affect dangerous motions for the ACL, especially in the soccer specific situations, in order to improve the effectivness of ACL injury prevention programs.

Traditionally, excessive knee extension, abduction, and internal-external rotation angles and moments during landing [6] are considered to be modifiable ACL injury risk factors. However, video analysis studies of noncontact ACL injuries found a significant impact of the ankle joint kinematics such as ankle dorsiflexion excursion angles at the time of injury on the risk of ACL injuries [7, 8]. One of the common problems among athletes is decreased ankle dorsiflexion range of motion (DF-ROM), which may result from injuries such as lateral ankle sprains [9]. Since the ankle and foot complex are in the lowest kinetic chain of the lower limbs, it appears that decreased ankle DF-ROM may affect the kinematics of the knee and hip during various landing tasks [10–12] and increase stiffness of landing [12], which in turn increase ACL injury risk [13].

While previous studies [10, 12, 14–17] have shown the impact of the ankle joint on poor movement patterns, however there is a limitation in these studies that the tasks used are controlled, i.e., the athlete’s attention is focused only on landing with no sport-specific movements. Indeed, movements at the time of occuring an ACL injury in the real world are often dual-task, i.e., the athlete’s attention is simultaneously focused on balls, goals, opponents, and other tasks [18, 19]. As such, it has been shown that the likelihood of increased load on the knee joint, and thus the risk of ACL injury, elevates when the dual task is performed during decelerating movements [20–23]. Furthurmore, it has been shown that people show different biomechanics that are considered to be associated with a higher ACL load and injury risk in the sport-specific tasks versus controlled ones [24]. With the best of our knowledge, we found no studies examining how ankle DF-ROM would affect movement patterns during a soccer-specific task.

The previous studies results are consist in laboratory-based measures; however they are gold standard, appear to pose a question on its external validity. To assess the movement patterns during landing in the soccer specific situation (landing with heading), we used a field-based and comprehensive multiplanar assessment, which attempts to consider all three planes of motion dysfunctions linked to increased ACL loading or injury risk [25].

Thus, the present study aimed to answer the question of whether there is a relationship between the clinical-based ankle DF-ROM measures and movement postures during a soccer-specific landing task, which better replicate the biomechanics of soccer athletes in the real-world environment. The hypothesis of the study was that decreased ankle DF-ROM would be associated with landing positions associated with noncontact ACL injury.

## Materials and methods

Thirty male soccer players from a professional soccer team U19 (aged between 16–19 years) with different playing positions (except the goalkeeper) participated in this descriptive study (mean ± SD: age = 16.8 ± 1.1 years, height = 1.7 ± 0.1 m, mass = 62.2 ± 10.4 kg, BMI = 20.3 ± 2.6 kg/m^2^, soccer playing experience = 6.9 ± 1.2 years). None of the participants had a previous ACL injury, lower extremity injury in the past 6 months such as ankle sprain, or lower extremity pain at the time of participation.

The soccer players were invited to the soccer field which aimed to introduce the research objectives and the procedures they would undergo. Their demographic data and sports-related injury history were collected using a brief questionnaire. Participants then signed a written informed consent form and completed a health status questionnaire. We also obtained written informed consent from parents or coaches on behalf of minors (< 18 years). The study was approved by the institutional research ethics committee. Before the study, participants warmed up with slow jogging and some dynamic stretching exercises, including double-leg squats.

### Ankle DF-ROM

**Fig 1:**
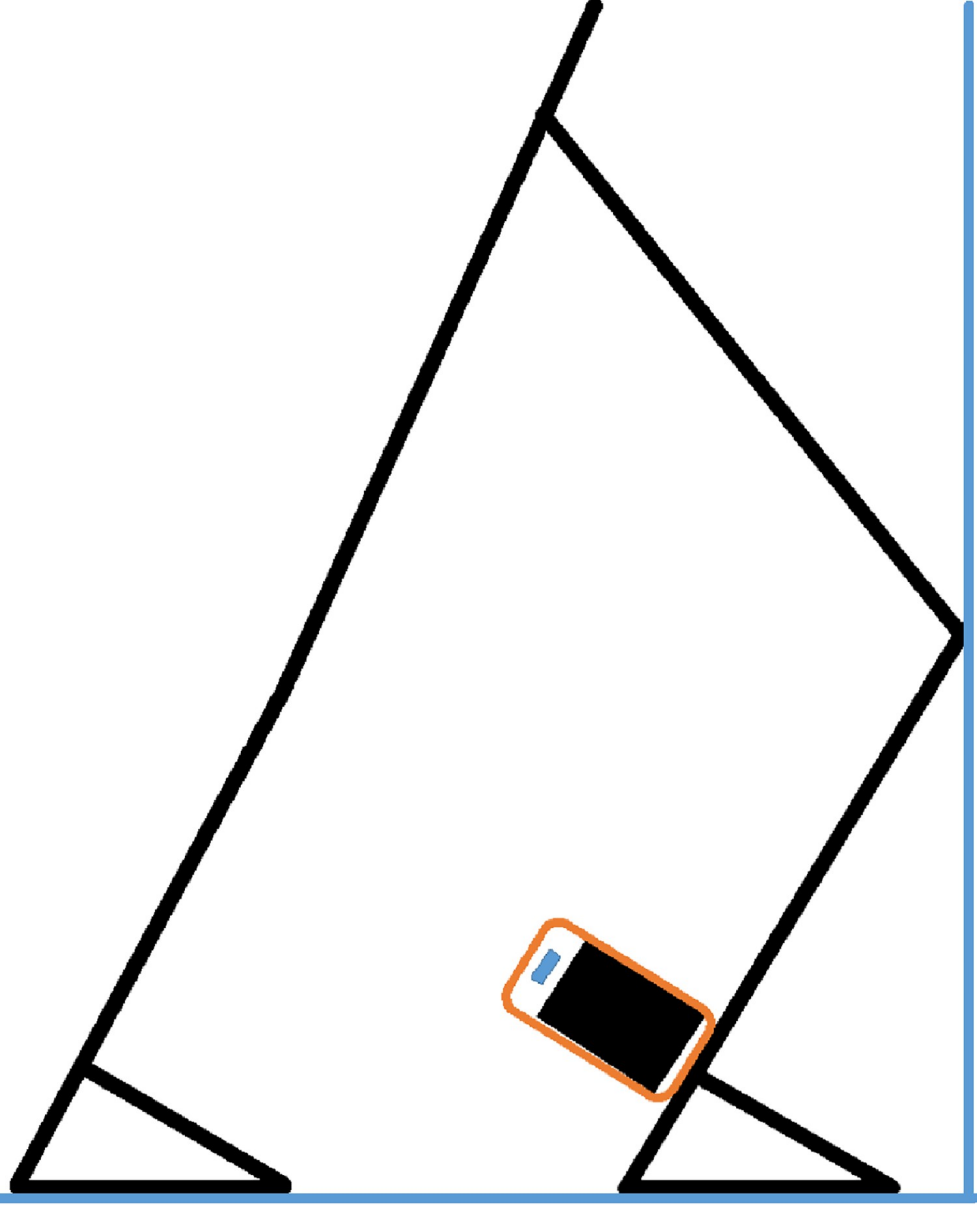
Position of weight-bearing lunge test (WBLT) demonstrated with Cell-Phone positioning.

The maximum ankle DF-ROM was measured by the tibial inclination angle using the weight-bearing lunge test (WBLT), a quick and convenient real-time measurement. This method has shown good inter-clinician (ICC = 0.80–0.99) and intra-clinician (ICC = 0.65–0.99) reliability [26]. The test was performed as described by Williams et al [27] with the participant positioned in front of the wall so that the dominant leg (preferred leg for kicking a ball for maximum distance) was perpendicular to the wall with the great toe. The participant’s knee was along the second toe and in line with the wall. The participant’s hands were against the wall and he was asked to slowly pull his foot back so that contact of the heel or knee with the wall could not be maintained. The cell phone was then placed on the posterior flat surface of the achilles tendon (approximately one centimeter superior to the posterior calcaneal tuberosity) and perpendicular to the shank of the tibia, and the ankle DF-ROM was measured with a digital inclinometer (TiltMeter APP) installed on the cell phone (Fig 1). This assessment was performed three times only for the dominant leg and the maximum value was used for analysis. The same examiner (H.A.) implemented all ankle DF-ROM measurements for all participants.

### Soccer-specific jump-landing task

**Fig 2:**
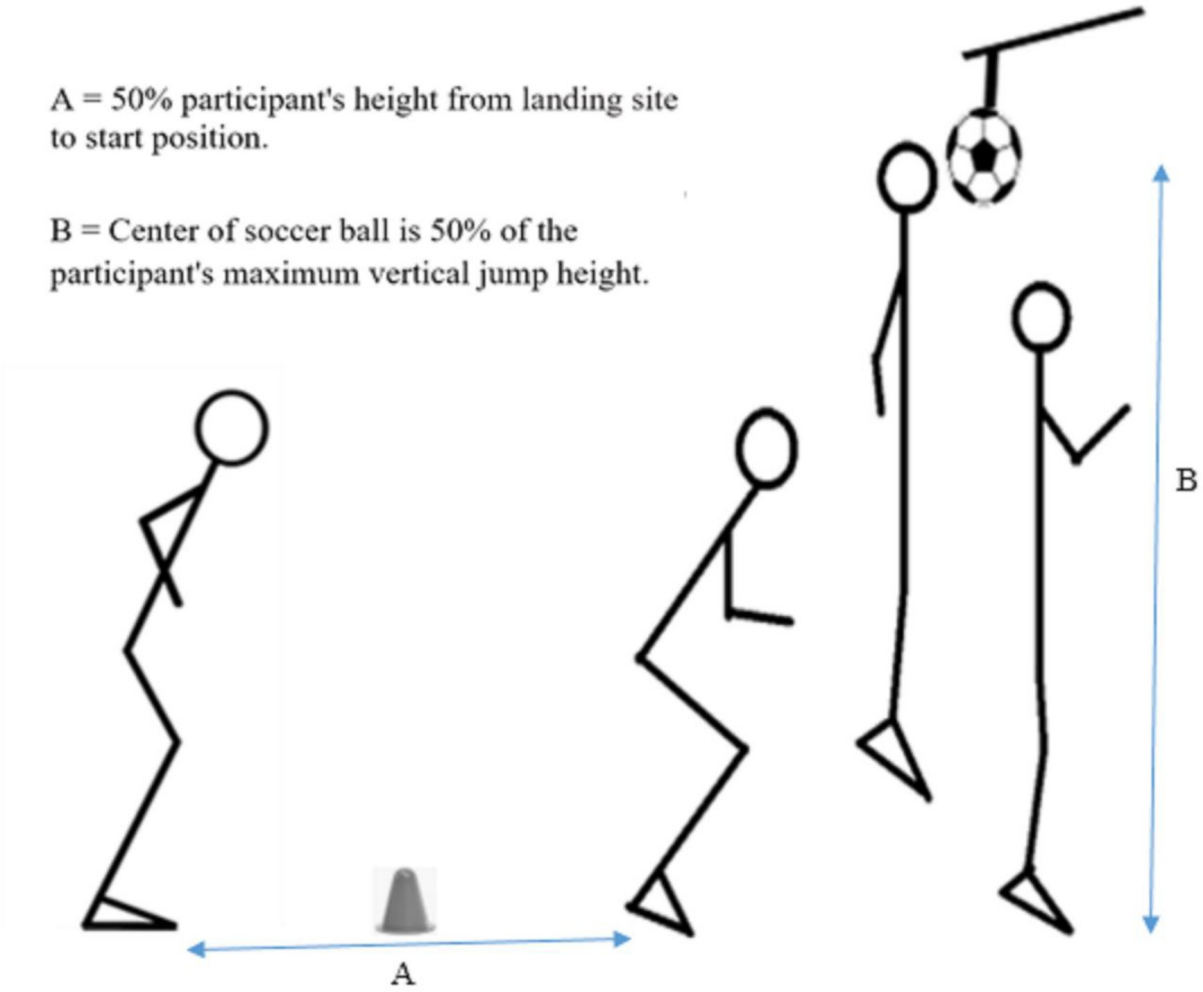
Soccer-specific jump-landing task.

The method for performing this test was presented by previously researchers [28]. In this test, the participant jumped from the ground in such a way that in order to reach the landing site (at a distance of half the participant’s height), the participant had to jump over a 7.5-centimeter cone (Fig 2). Immediately after landing, participants were instructed to jump vertically and head the soccer ball, which was attached to a point equal to 50% of the participant’s maximum vertical jump height. After being instructed in the task, participants were allowed to practice it twice in order to perform it correctly. Two digital video cameras (Panasonic Lumix DMC-FZ200, Japan) were positioned 3 m in front of and to the right of the participants to record all jump landing attempts. The test was performed on the soccer field (natural grass) while the participants wore their own soccer footwear. They performed 3 successful trials of this task.

It is worth noting that before task performance and after warm-up, the height of the maximum vertical jump was determined for each participant. The evaluation of the maximum vertical jump was performed as follows: First, the participants stood next to the wall, made a mark on the wall with one hand, and then jumped up with maximum effort and made another mark on the wall with the same hand. The difference between these two values was taken as the participant’s maximum vertical jump. A maximum of three jumps were considered and half of them were used to determine the height of the ball.

After data collection, the landing technique was evaluated using Kinovea software (version 0.8.15, Kinovea Open Source Project, www.kinovea.org) and video scoring was based on the items in Table 1, which was adapted from the landing error scoring system (LESS), originally developed by Padua et al [25]. LESS assesses 17 items on a scale from 0–19. A higher score (greater number of landing errors) indicates potentially high-risk movement patterns [25]. The landing of all participants was quantified by a trained LESS rater (H.A.). The movements of the lower extremities and trunk were analyzed at the moment of "initial contact of the foot with the ground" and "between initial contact of the foot with the ground and maximum knee flexion" and only for the right foot to simplify the evaluation. To analyze the overall landing technique, the mean of the scores from the 3 jump landing trials was calculated.

**Table 1:** LESS items (scoring: 0 = best to 19 = worse landing quality).

LESS items	Operational definition^a^	Camera view	LESS score
1. Knee flexion angle at initial contact	At the time point of initial contact, if the knee of the test leg is flexed more than 30 degrees, score YES. If the knee is not flexed more than 30 degrees, score NO.	Side	Yes = 0 No = 1
2. Hip flexion angle at initial contact	At the time point of initial contact, if the thigh of the test leg is in line with the trunk then the hips are not flexed and score NO. If the thigh of the test leg is flexed on the trunk, score YES.	Side	Yes = 0 No = 1
3. Trunk flexion angle at initial contact	At the time point of initial contact, if the trunk is vertical or extended on the hips, score NO. If the trunk is flexed on the hips, score YES.	Side	Yes = 0 No = 1
4. Ankle plantarflexion angle at initial contact	If the foot of the test leg lands toe to heel, score YES. If the foot of the test leg lands heel to toe or with a flat foot, score NO	Side	Yes = 0 No = 1
5. Knee valgus angle at initial contact	At the time point of initial contact, draw a line straight down from the center of the patella. If the line goes through the midfoot, score NO. If the line is medial to the midfoot, score YES.	Frontal	Yes = 1 No = 0
6. Lateral trunk flexion angle at initial contact	At the time point of initial contact, if the midline of the trunk is flexed to the left or the right side of the body, score YES. If the trunk is not flexed to the left or right side of the body, score NO.	Frontal	Yes = 1 No = 0
7. Stance width–Wide	Once the entire foot is in contact with the ground, draw a line down from the tip of the shoulders. If the line on the side of the test leg is inside the foot of the test leg then greater than shoulder width (wide), score YES. If the test foot is internally or externally rotated, grade the stance width based on heel placement.	Frontal	Yes = 1 No = 0
8. Stance width–Narrow	Once the entire foot is in contact with the ground, draw a line down from the tip of the shoulders. If the line on the side of the test leg is outside of the foot then score less than shoulder width (narrow), score YES. If the test foot is internally or externally rotated, grade the stance width based on heel placement.	Frontal	Yes = 1 No = 0
9. Foot position–Toe in	If the foot of the test leg is internally more than 30 degrees between the time period of initial contact and max knee flexion, then score YES. If the foot is not internally rotated more than 30 degrees between the time period of initial contact to max knee flexion, score NO.	Frontal	Yes = 1 No = 0
10. Foot position–Toe out	If the foot of the test leg is externally rotated more than 30 degrees between the time period of initial contact and max knee flexion, then score YES. If the foot is not externally rotated more than 30 degrees between the time period of initial contact to max knee flexion, score NO.	Frontal	Yes = 1 No = 0
11. Symmetric initial foot contact	If one foot lands before the other or if one foot lands heel to toe and the other lands toe to heel, score NO. If the feet land symmetrically, score YES.	Frontal	Yes = 0 No = 1
12. Knee flexion displacement	If the knee of the test leg flexes more than 45 degrees from initial contact to max knee flexion, score YES. If the knee of the test leg does not flex more than 45 degrees, score NO.	Side	Yes = 0 No = 1
13. Hip flexion at max knee flexion	If the thigh of the test leg flexes more on the trunk from initial contact to max knee flexion angle, score YES.	Side	Yes = 0 No = 1
14. Trunk flexion at max knee flexion	If the trunk flexes more from the point of initial contact to max knee flexion, score YES. If the trunk does not flex more, score NO.	Side	Yes = 0 No = 1
15. Knee valgus displacement	At the point of max knee valgus on the test leg, draw a line straight down from the center of the patella. If the line runs through the great toe or is medial to the great toe, score YES. If the line is lateral to the great toe, score NO.	Frontal	Yes = 1 No = 0
16. Joint displacement	Watch the sagittal plane motion at the hips and knees from initial contact to max knee flexion angle. If the participant goes through large displacement of the trunk, hips, and knees, then score SOFT. If the participant goes through some trunk, hip, and knee displacement but not a large amount, then AVERAGE. If the participant goes through very little, if any trunk, hip, and knee displacement, then STIFF.	Side	Soft = 0 Av. = 1 Stiff = 2
17. Overall impression	Score EXCELLENT if the participant displays a soft landing and no frontal plane motion at the knee, Score POOR if the participant displays a stiff landing and large frontal plane motion at the knee. All other landings, score AVERAGE.	Side, Frontal	Ex. = 0 Av. = 1 Poor = 2

**Abbreviations:** LESS, landing error scoring system; Av., average; Ex., excellent. ^a^From Padua et al (2009) [25]

### Statistical analyses

Data were analyzed using IBM SPSS Statistics for Windows (version 25.0; IBM Corp, Armonk, NY). The distribution of quantitative data was assessed with the Shapiro-Wilk test (P > .05). For the main objective of the study, the Pearson correlation test (*r*) was used to analyze the relationship between ankle DF-ROM and landing quality scores during the soccer-specific task. The *r* values are distributed as follows: r = 0.10–0.29, small or low correlation; r = 0.30–0.49, medium or moderate correlation; r = 0.50–1.0, large or high or strong correlation [29]. Statistical significance was set at p < 0.05.

## Results

**Fig 3:**
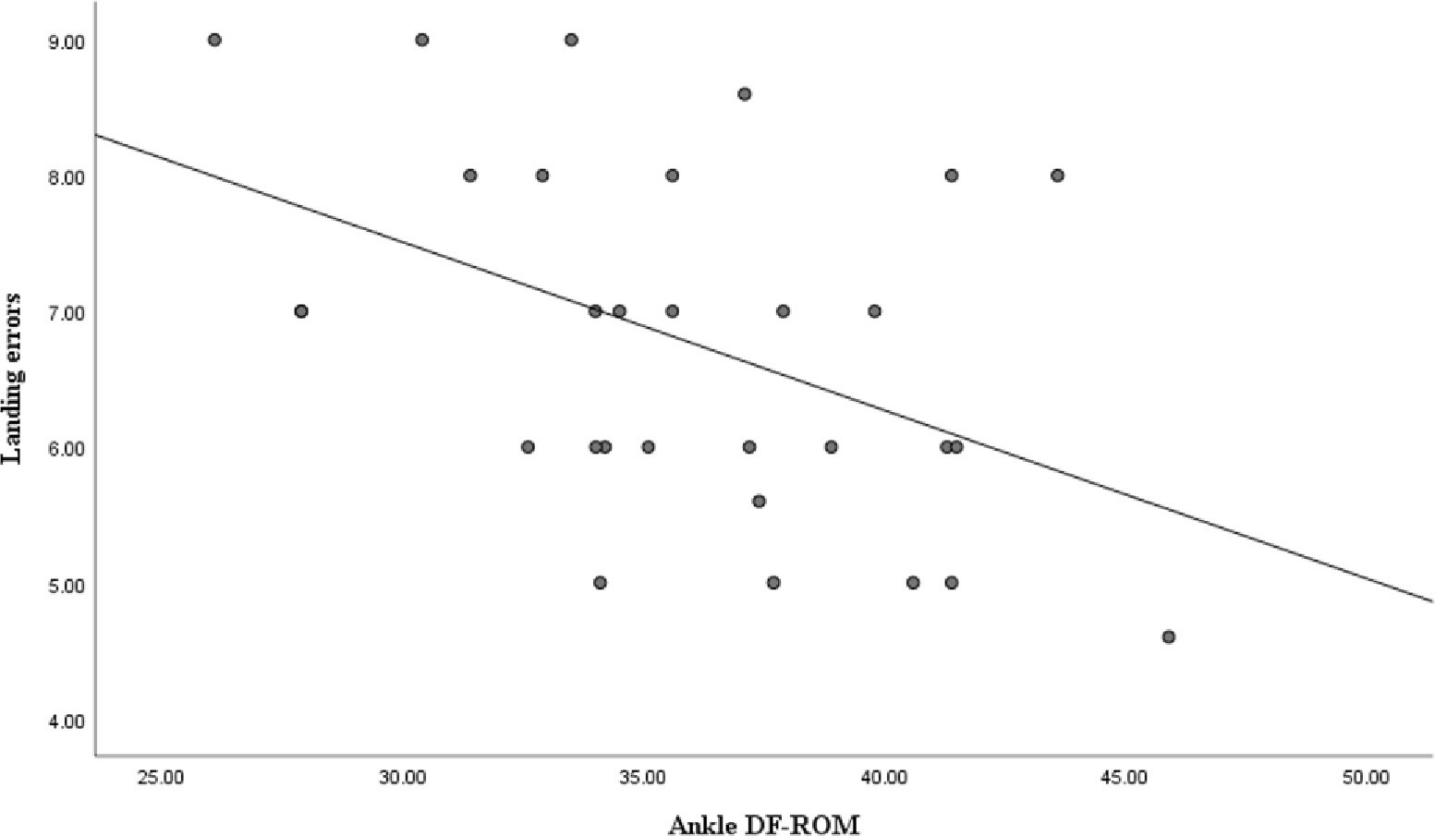
Scatter diagram of ankle dorsiflexion range of motion (DF-ROM) and landing errors.

As shown in Table 2 and Fig 3, the results of the Pearson correlation test indicated that there is a negative relationship between the scores related to landing kinematics in the soccer-specific jump landing task (range = 4.6–9.0, mean ± SD = 6.8 ± 1.3) and the ankle DF-ROM scores (range = 26.1–45.9, mean ± SD = 36.1 ± 4.8) (r = -0.450, P = 0.006). This means that soccer players with decreased ankle DF-ROM have more landing errors in the soccer-specific task. The obtained correlation strength is moderate and 20.3% of the variation in soccer-specific jump landing performance could be attributed to the values of the ankle DF-ROM.

**Table 2:** The results of Pearson correlation test.

Variable	Mean ± SD	Range	r	r^2^	P
Soccer-specific landing score	6.8 ± 1.3	4.6–9.0	-0.450	0.203	0.006*
Ankle DF-ROM (°)	36.1 ± 4.8	26.1–45.9			

**Abbreviations:** *, Correlation is significant at the 0.01 level (1-tailed); DF-ROM, dorsiflexion range of motion.

## Discussion

The current study aimed to investigate the relationship between the ankle DF-ROM and specific movement patterns that normally occur in ACL injury during landing with heading. The results of this study showed that decreasing the ankle DF-ROM could place the lower extremity in a position where the athlete is at higher risk for ACL injury. Scoring in the soccer-specific task is designed to identify potentially high-risk movement patterns associated with noncontact ACL injuries as errors in the organized execution of a jump-landing task. Scores are based on observable jump landing errors, and a high score indicates poor technique and consequently greater risk for noncontact ACL injury [25]. Our hypothesis was supported by our results, as we found that a decreased ankle DF-ROM is associated with more landing errors and therefore may places the lower extremity in a position to withstand greater ACL loading, which in turn may increases the risk of noncontact ACL injury. Although the strength of the correlation was moderate, with the knowledge available, this study is the first study to examine the relationship between the ankle DF-ROM and specific movement patterns associated with noncontact ACL injury when landing on a soccer-specific task. These results are consistent with studies that have shown decreased DF-ROM could negatively affect on movement patterns [10, 12, 14–17]. For example, Taylor et al in their study on female athletes indicated that limited ankle DF-ROM is associated with movement dysfunction that may lead to a higher risk of sustaining injury such as lower levels of knee and hip flexion and hip and knee extensor work absorption and aberrant frontal plane knee motion [17]. Hagins et al examined the effects of DF-ROM reduction on landing mechanics and found that DF-ROM reduction was associated with greater valgus displacement of the knee and increased ground reaction forces (GRFs) [14]. The study by Fong et al showed that a decrease in DF-ROM, measured in a extended-knee position (measured with a goniometer), was related to a decrease in knee flexion displacement and an increase in vertical and posterior GRFs on landing [12]. Although they did not find this relationship for the amount of DF-ROM measured in the flexed knee position, the results were close to significant for these variables. In addition, Sigward et al in a study on high school soccer players reported that a reduction in passive DF-ROM (measured with a goniometer with the knee flexed at 30 degrees) increased knee abduction on landing [15]. Dowling et al showed that DF-ROM and maximum hip flexion on landing were positively associated with each other [16]. The results of our study were not consistent with the results of a study by Dill et al conducted on 40 physically active participants [11]. In their study, the researchers divided the participants into two groups, one normal and one limited DF-ROM, and examined DF-ROM in two positions with the knee extended and without weight bearing and with weight bearing by WBLT. In this study, they found no difference between the different DF-ROM groups in terms of knee flexion displacement, ankle dorsiflexion, and maximal knee flexion during jump landing; however, in overhead and single-leg squats, there was a difference between the groups in terms of these variables. Both the methodology and the biomechanical measurement devices used in this study (motion analysis system) differed from those used in the present study.

In our study, participants who had decreased ankle DF-ROM had higher landing errors, implied that the lower the ankle DF-ROM, the greater pathomechanical landing characteristics related to noncontact ACL injuries. The ankle plays an important role in absorbing and dissipating GRFs during landing, although all lower limb joints (ankle, knee, and hip) must work together to absorb landing forces [30]. Sagittal plane coupling theory states that increased/decreased flexion in one joint is associated with a concomitant increase/decrease in flexion in other lower limb joints during landing [30, 31]. Furthermore, by decreasing the ankle dorsiflexion, the ability of the triceps surae to absorb GRFs decreases, and GRFs are transferred to the upper joints [32, 33]. Higher GRFs have been shown to be associated with greater valgus displacement of the knee [33]. When the triceps surae is unable to absorb GRFs, the hamstring muscles cannot flex the knee to absorb impact forces [32]. Low flexion of the knee during landing increases the anterior tibial shear force, which places a high strain on the ACL and increases the risk of injury [6]. Therefore, less dorsiflexion of the ankle could contribute to less displacement of knee flexion, resulting in a stiffer landing strategy and increasing GRF, valgus displacement of the knee and anterior tibial shear force.

Understanding the mechanism and identifying risk factors for injury are critical to designing the components of a prevention program [25, 34–36]. Research on risk factors for injury is supported for two reasons: first, to understand why injuries occur, and second, to predict who is at risk for injury, although there are some difficulties in achieving this goal [37]. Neuromuscular factors associated with noncontact ACL injuries can be modified by appropriate screening methods and training programs [38]. Therefore, neuromuscular control of the knee may reduce the risk of ACL injury. Movement screening tasks are often introduced to identify high-risk athletes. Previous studies have shown that the effectiveness of injury prevention programs is better when they target athletes who are at higher risk for ACL injury [39, 40]. The landing evaluation system in this study is based on 17 movement characteristics during landing [25]. In this scoring system, specific movement patterns typically seen in ACL injury, such as knee valgus, excessive tibial rotation, reduced knee and hip flexion, poor trunk control, and lower limb asymmetry on landing, are examined in an orderly and simple manner. The scores obtained with this evaluation system are based on the errors during the jump landing. A high score indicates poor technique and therefore a higher risk to the lower limb. One of the main features of this scoring system that distinguishes it from other tools is the analysis of landing technique in all three planes of motion. Assessment of landing motion in multiple planes is important because ACL injuries are thought to occur with complex multidimensional motion [6, 41]. The findings of this study demonstrate that decreasing the ankle DF-ROM could place the lower extremity in a position where the athlete is at higher risk for ACL injury. To reduce the potentially high-risk biomechanical movement patterns for ACL injury, it may be useful to include exercises to increase DF-ROM in the prevention program. Some evidence exists to support the efficacy of stretching alone and stretching in combination with other prevention programs in increasing ankle joint. For example, the lunge exercise included in the FIFA 11+ program found beneficial effects for ankle flexibility [42].

In this study, soccer-specific jump-landing task was performed on the soccer field (natural grass) while the participants wore their own soccer footwear, which would increase the generalizability of the results under real conditions. Furthermore, the evaluation of landing motion was performed using LESS, a field-based functional movement assessment tool that is easy to administer and requires minimal equipment and time (total set-up time per participant is less than 5 min). The ankle DF-ROM was also measured by using the WBLT, a clinical test that is quick (time per participant is less than 1 min) and convenient real-time measurement. However, it should be noted that when using the results of the present study, its limitations should also be considered. One of the limitations of this study was that it was not possible to equalize the jump height of all participants in the soccer task. However, by using a 7.5-cm cone between the jump site and the participant, this limitation can probably be reduced to some degree. Furthermore, our participants were chosen from among the young male professional soccer players aged 16 to 19 years. Thus, these results may not be applicable to other population with different chractristics [43]. Therefore, it is suggested that studies be conducted for other populations, such as adults and adolescents, other activity levels and especially for female soccer players. It is also proposed to investigate the ability of soccer-specific task and decreased ankle DF-ROM in predicting ACL injuries in soccer.

## Conclusion

Our results suggested that faulty landing motion may be caused by decreased ankle DF-ROM. Thus, adding ankle DF-ROM measurement in screening process may help identifying underlining cause of the faulty motion and correcting athlete’s risky motion.
